# Estimating the Prevalence of Transparency and Reproducibility-Related Research Practices in Psychology (2014–2017)

**DOI:** 10.1177/1745691620979806

**Published:** 2021-03-08

**Authors:** Tom E. Hardwicke, Robert T. Thibault, Jessica E. Kosie, Joshua D. Wallach, Mallory C. Kidwell, John P. A. Ioannidis

**Affiliations:** 1 Department of Psychology, University of Amsterdam; 2 Meta-Research Innovation Center Berlin (METRIC-B), QUEST Center for Transforming Biomedical Research, Berlin Institute of Health, Charité–Universitätsmedizin Berlin; 3 School of Psychological Science, University of Bristol; 4 MRC Integrative Epidemiology Unit at the University of Bristol; 5 Department of Psychology, Princeton University; 6 Department of Environmental Health Sciences, Yale School of Public Health; 7 Department of Psychology, University of Utah; 8 Department of Medicine, Stanford University; 9 Department of Epidemiology and Population Health, Stanford University; 10 Department of Biomedical Data Science, Stanford University; 11 Department of Statistics, Stanford University; 12 Meta-Research Innovation Center at Stanford, Stanford University

**Keywords:** transparency, reproducibility, meta-research, psychology, open science

## Abstract

Psychologists are navigating an unprecedented period of introspection about the credibility and utility of their discipline. Reform initiatives emphasize the benefits of transparency and reproducibility-related research practices; however, adoption across the psychology literature is unknown. Estimating the prevalence of such practices will help to gauge the collective impact of reform initiatives, track progress over time, and calibrate future efforts. To this end, we manually examined a random sample of 250 psychology articles published between 2014 and 2017. Over half of the articles were publicly available (154/237, 65%, 95% confidence interval [CI] = [59%, 71%]); however, sharing of research materials (26/183; 14%, 95% CI = [10%, 19%]), study protocols (0/188; 0%, 95% CI = [0%, 1%]), raw data (4/188; 2%, 95% CI = [1%, 4%]), and analysis scripts (1/188; 1%, 95% CI = [0%, 1%]) was rare. Preregistration was also uncommon (5/188; 3%, 95% CI = [1%, 5%]). Many articles included a funding disclosure statement (142/228; 62%, 95% CI = [56%, 69%]), but conflict-of-interest statements were less common (88/228; 39%, 95% CI = [32%, 45%]). Replication studies were rare (10/188; 5%, 95% CI = [3%, 8%]), and few studies were included in systematic reviews (21/183; 11%, 95% CI = [8%, 16%]) or meta-analyses (12/183; 7%, 95% CI = [4%, 10%]). Overall, the results suggest that transparency and reproducibility-related research practices were far from routine. These findings establish baseline prevalence estimates against which future progress toward increasing the credibility and utility of psychology research can be compared.

Serious concerns about the credibility and utility of some scientific research (Ioannidis, 2005; Ioannidis, Greenland, et al., 2014) have prompted calls for increasingly adopting research practices that enhance reproducibility and transparency (Miguel et al., 2014; Munafò et al., 2017; Nosek et al., 2015; Wallach, Gonsalves, & Ross, 2018). Close scrutiny of psychology in particular has suggested that standard research and publication practices have rendered the discipline highly exposed to bias, potentially resulting in a large volume of exaggerated and misleading results (Ioannidis, Munafò, et al., 2014; John et al., 2012; Open Science Collaboration, 2015; Pashler & Wagenmakers, 2012; Simmons et al., 2011; Szucs & Ioannidis, 2017). This realization has led to a number of reform efforts (Hardwicke & Ioannidis, 2018a; Hardwicke, Serghiou, et al., 2020; Nelson et al., 2018; Vazire, 2018) that have the potential to improve efficiency (Chalmers & Glasziou, 2009), facilitate self-correction (Ioannidis, 2012), and enhance credibility (Vazire, 2017). To gauge the collective impact of these efforts, track progress over time, and calibrate future policy and training initiatives, it will be useful to assess the prevalence of various transparency and reproducibility-related practices across the field of psychology.

A central focus of reform initiatives has been to encourage scientists to share more information about the studies they perform. Journal articles are only the most visible facade of deeper layers of scholarship that may include protocols, original research materials, raw data, and analysis scripts—resources that are not necessarily shared with other scientists (Buckheit & Donoho, 1995; Klein et al., 2018). Even journal articles themselves may only be accessible to those with institutional access or the ability to pay a fee (Piwowar et al., 2018). In addition, potential sources of bias, such as conflicts of interest and funding sources, may not be disclosed (Bekelman et al., 2003; Cristea & Ioannidis, 2018). However, there is a growing (or reemerging; David, 2008) appreciation that the scientific community needs to be able to access all of this information to comprehensively evaluate, interpret, and independently verify scientific claims (Munafò et al., 2017; Vazire, 2017). Furthermore, access to this information enables replication, evidence synthesis, and discovery activities that may ultimately accelerate scientific progress (Ioannidis, 2012; Klein et al., 2018).

The burgeoning discipline of meta-research (“research on research”) has already begun to evaluate the impact of various reform initiatives (Hardwicke, Serghiou, et al., 2020). For example, journal data-sharing policies have been associated with moderate to substantial increases in data sharing (Hardwicke et al., 2018; Kidwell et al., 2016; Nuijten et al., 2017; Rowhani-Farid & Barnett, 2016; Naudet et al., 2018). However, the prevalence of transparency and reproducibility-related research practices in psychological science is largely unknown. Using previous investigations in biomedicine (Iqbal et al., 2016; Wallach et al., 2018) and the social sciences (Hardwicke, Wallach, et al., 2020) as a guide, we manually examined a random sample of 250 articles to estimate the prevalence of several transparency and reproducibility-related indicators in psychology articles published between 2014 and 2017. The indicators were open access to published articles; availability of study materials, study protocols,^1^ raw data, and analysis scripts; preregistration; disclosure of funding sources and conflicts of interest; conduct of replication studies; and cumulative synthesis of evidence in meta-analyses and systematic reviews.

## Method

### Design

This was a retrospective observational study with a cross-sectional design. Sampling units were individual articles. Measured variables are shown in Table 1.

**Table 1.** Measured Variables

Variable	Applicable study designs
Article characteristics	
Subject area, year of publication, study design, country of origin (based on corresponding author’s affiliation), human/animal subjects, 2017 journal impact factor (according to Thomson Reuters Journal Citation Reports)	All
Articles	
Accessibility and retrieval method (can the article be accessed, and, if so, is there a public version or is paywall access required?)	All
Protocols	
Availability statement (is availability, or lack of, explicitly declared?)	
Content (what aspects of the study are included in the protocol?)	
Materials	
Availability statement (is availability, or lack of, explicitly declared?)	Study designs involving primary data^a^
Retrieval method (e.g., on request or via online repository)	
Accessibility (can the materials be accessed?)	
Raw data	
Availability statement (is availability, or lack of, explicitly declared?)	Study designs involving primary data,^a^ study designs involving secondary data (commentaries with analysis and meta-analyses)
Retrieval method (e.g., on request or via online repository)	
Accessibility (can the data be accessed?)	
Content (have all relevant data been shared?)	
Documentation (are the data understandable?)	
Analysis scripts	
Availability statement (is availability, or lack of, explicitly declared?)	
Retrieval method (e.g., on request or via online repository)	
Accessibility (can the scripts be accessed?)	
Preregistration	
Availability statement (is availability, or lack of, explicitly declared?)	Study designs involving primary data,^a^ study designs involving secondary data (commentaries with analysis and meta-analyses)
Retrieval method (which registry was used?)	
Accessibility (can the preregistration be accessed?)	
Content (what was preregistered?)	
Funding	
Disclosure statement (are funding sources, or lack of, explicitly declared?)	All
Conflicts of interest	
Disclosure statement (are conflicts of interest, or lack of, explicitly declared?)	All
Replication	
Statement (does the article claim to report a replication?)	All
Citation history (has the article been cited by a study that claims to be a replication?)	Study designs involving primary data^a^
Evidence synthesis	
Meta-analysis citation history^b^ (has the article been cited by and included in the evidence-synthesis component of a meta-analysis?)	Study designs involving primary data^a^
Systematic review citation history^b^ (has the article been cited by and included in the evidence-synthesis component of a systematic review?)	Study designs involving primary data^a^

Note: The variables measured for an individual article depended on the study-design classification. For articles that were not available (the full text could not be retrieved) or were not in English, only article characteristics were obtained. The exact operational definitions and procedures for data extraction/coding are available in the structured form at https://osf.io/x9rmy/yh.

a Encompasses the following study-design classifications: field studies, laboratory studies, surveys, case studies, multiple types, clinical trial, and other designs. ^b^Meta-analysis and systematic review variables were coded as exclusive variables.

The study protocol was preregistered on September 26, 2018, and is available at https://osf.io/q96eh. All deviations from this protocol are explicitly acknowledged in Section A of the Supplemental Material available online. We report how we determined our sample size, all data exclusions, all manipulations, and all measures in the study. All data (https://osf.io/5qmz7), materials (https://osf.io/c89sy), and analysis scripts (https://osf.io/gfjtq) related to this study are publicly available. To facilitate reproducibility, the manuscript was written by interleaving regular prose with analysis code and is available in a Code Ocean container (https://doi.org/10.24433/CO.1618143.v2) that recreates the software environment in which the original analyses were performed.

### Sample

We obtained a random sample of 250 psychology articles published between 2014 and 2017. We used a random-number generator to sample 250 articles from all 224,556 documents in the Scopus database (as of September 22, 2018) designated with the document type “article” or “review” and with an All Science Journal Classification (ASJC) code related to psychology (i.e., ASJC codes 3200–3207). This classification included 1,323 journals and all subject areas shown in Table 2. The sample size was based on our judgment of what was both informative and tractable. The evaluated time period was selected to represent recently published articles (relative to the start of data collection) and to facilitate comparisons with a similar assessment conducted in the social sciences for the same time period (Hardwicke, Wallach, et al., 2020).

**Table 2.** Sample Characteristics for the 250 Randomly Sampled Articles and the 228 English-Language and Accessible Articles That Were Eligible for In-Depth Data Extraction

Characteristic	Eligible articles (*n*)	All articles (*n*)
Subject area		
Clinical psychology	57	65
General psychology	41	43
Developmental and educational psychology	40	44
Applied psychology	34	38
Social psychology	29	33
Experimental and cognitive psychology	16	16
Neuropsychology and physiological psychology	10	10
Psychology (miscellaneous)	1	1
Year of publication		
2014	54	58
2015	60	68
2016	49	54
2017	65	70
Study design		
Survey/interview	73	77
Laboratory study	52	53
No empirical data	40	48
Observational study	33	40
Clinical trial	15	15
Multiple study types	6	6
Case study	4	4
Commentary with analysis	3	3
Meta-analysis	2	2
Country of origin		
United States of America	99	99
United Kingdom	20	20
Canada	12	13
Germany	11	15
The Netherlands	11	11
28 other countries^a^ (accounting for < 10 per country)	75	92
Subjects		
Humans	174	184
Animals	7	7
Both	1	1
Neither humans nor animals involved	46	56

Note: The 2017 median impact factor for eligible articles was 2.23 (range = 0.33–15.07); the median impact factor for all articles was 2.06 (range = 0.22-15.07). The publication-year median impact factor for eligible articles was 2.09 (range = 0.23-20.77); the publication-year median impact factor for all articles was 2.00 (range = 0.22–20.77). No 2017 journal impact factor was available for 111 articles (97 eligible articles), and no publication-year impact factor was available for 113 articles (99 eligible articles).

a For all countries, see https://osf.io/kg7j5.

### Procedure

Data were collected between September 27, 2018, and September 23, 2019. Data extraction for the measured variables shown in Table 1 involved close manual examination of the articles supplemented with keyword searching guided by a Google form (https://osf.io/x9rmy) based on previous investigations in biomedicine (Iqbal et al., 2016; Wallach et al., 2018) and the social sciences (Hardwicke, Wallach, et al., 2020). As indicated in the second column of Table 1, the exact variables measured for a given article depended on the study design. Attempts to access each article were made by searching with the Open Access Button, Google, and, if necessary, at least two of the online libraries of the coders’ affiliated universities. For articles that were not available (the full text could not be retrieved) or were not published in English, only article characteristics were obtained.

Three investigators performed the initial extraction and coding; articles were assigned to each investigator on demand by randomly selecting from the remaining articles (J. D. Wallach, *n* = 32; J. E. Kosie, *n* = 93; R. T. Thibault, *n* = 125). A single investigator obtained the subject area, year of publication, and citation histories from Scopus and 2017 journal impact factors from Thomson Reuters Journal Citation Reports. All other variables in Table 1 were coded a second time by one of two other investigators (T. E. Hardwicke, *n* = 198; M. C. Kidwell, *n* = 52), and any coding differences were resolved through discussion. Interrater reliability assessments are shown in Table S1 in the Supplemental Material available online. Assuming all articles were published on July 1 of their respective publication year (i.e., halfway through the year), the time between publication and recording citation information ranged from 448 to 1,544 (*Mdn* = 996) days for number of citations and 456 to 1,900 (*Mdn* = 1,178) days for number of citing articles that were replications, systematic reviews, and/or meta-analyses. Conflict-of-interest statements (Table 3) were categorized by two investigators (T. E. Hardwicke and J. D. Wallach) in an exploratory manner (i.e., not preregistered).

**Table 3.** Frequency of Different Types of Conflict of Interest Reported in the 12 Statements Reporting One or More Conflicts of Interest

Type of conflict of interest	Frequency of appearance in statements (*n*)
Industry-related	
Authorship/editorship royalties	4
Research funding from industry	4
Served on industry advisory board	4
Consultancy for industry	3
Ownership of relevant commercial products, patents, or procedures	3
Speaking fees from industry	3
Employed by industry	2
Honoraria from industry	2
Industry equity holder	2
Travel or hospitality awards from industry	2
Other undefined payments from industry	1
Nonindustry-related	
Research funding from government	4
Research funding from foundations, charities, and/or NGOs	2
Consultancy for foundations, charities, and/or NGOs	1
Honoraria from foundations, charities, and/or NGOs	1

Note: Because each of the 12 relevant conflict-of-interest statements may contain more than one type of conflict of interest, the frequency column sums to greater than 12. NGO = nongovernmental organization.

### Analysis

Results are descriptive and focus on the proportion of articles that fulfill each of the evaluated indicators, using as a denominator the number of articles in which each indicator is applicable (see Table 1). We also report 95% confidence intervals (CIs) based on the Sison-Glaz method for multinomial proportions (Sison & Glaz, 1995).

## Results

### Sample characteristics

Sample characteristics for all 250 articles and for the 228 articles that were eligible for in-depth data extraction (i.e., in English and accessible) are displayed in Table 2.

### Article availability (open access)

**Fig. 1.**
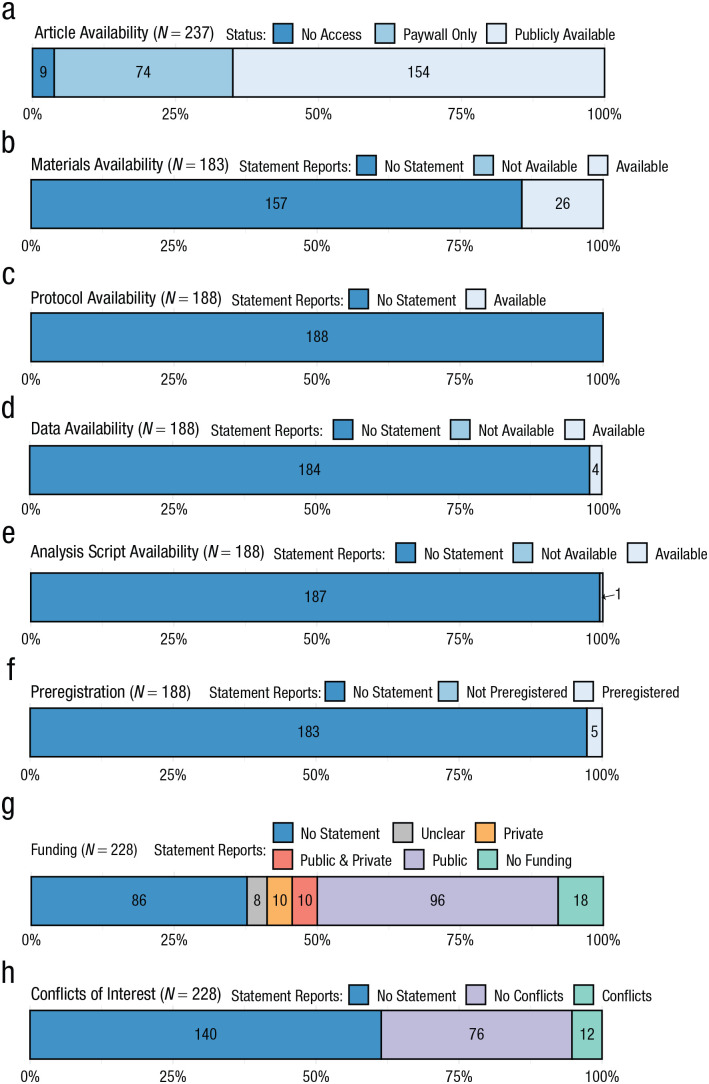
Assessment of transparency and reproducibility-related research practices in psychology. The *x*-axis shows the percentage of the total number of articles (*N*) assessed for a given indicator (which was contingent on the study design; see Table 1). Raw counts are shown inside bars.

Among the 237 English-language articles, we obtained a publicly available version for 154 (65%, 95% CI = [59%, 71%]; Fig. 1a), whereas 74 (31%, 95% CI = [25%, 38%]) were only accessible through a paywall. Nine articles (4%, 95% CI = [0%, 10%]) were not available at all (Fig. 1a).

### Materials and protocol availability

Of the 183 articles that involved primary data (see Table 1), 26 contained a statement regarding the availability of original research materials such as survey instruments, software, or stimuli (14%, 95% CI = [10%, 19%]; Fig. 1b). Of the 188 articles involving primary or secondary data, zero reported the availability of a study protocol (0%, 95% CI = [0%, 1%]; Fig. 1c).

For the 26 articles for which materials were reportedly available, the materials were not actually available for seven articles because of broken links. For the 19 articles that we could access, the materials were made available in the article itself (e.g., in a table or appendix; *n* = 8), in a journal-hosted supplement (*n* = 6), on a personal or institutionally hosted (nonrepository) webpage (*n* = 3), or in an online third-party repository (*n* = 2).

### Data availability

Of the 188 articles that involved primary or secondary data, four contained data-availability statements (2%, 95% CI = [1%, 4%]; Fig. 1d). For one data set, a fee was required (which we did not pay) to obtain access. Of the three accessible data sets, two were available via an online third-party repository, and one was available in journal-hosted supplemental materials. One data set was incomplete, whereas the remaining two appeared complete and clearly documented.

### Analysis-script availability

Of the 188 articles that involved primary or secondary data, an analysis script was shared for one article (1/188; 1%, 95% CI = [0%, 1%]; Fig. 1e) via a third-party repository.

### Preregistration

Of the 188 articles involving primary or secondary data, five included a statement regarding preregistration (3%, 95% CI = [1%, 5%]; Fig. 1f). One preregistration at EUDRA-CT (European Union Drug Regulating Authorities Clinical Trials Database) was not accessible. The accessible preregistrations were from ClinicalTrials.gov (*n* = 2) and the Netherlands Trials Register (*n* = 2) and pertained to three clinical trials and one observational study. All accessible preregistrations contained information about hypotheses and methods but did not contain analysis plans.

### Funding and conflict-of-interest statements

Of the 228 English-language and accessible articles, 142 included a statement about funding sources (62%, 95% CI = [56%, 69%]; Fig. 1g). Most articles disclosed public funding only (*n* = 96; 42%), 10 (4%) disclosed private funding only, 10 (4%) disclosed a combination of public and private funding, and 18 (8%) disclosed that the work had received no funding. We could not clearly determine the status of the funding for eight articles.

Eighty-eight of the 228 articles included a conflicts-of-interest statement (39%, 95% CI = [32%, 45%]; Fig. 1h). Of these 88 articles, most reported that there were no conflicts of interest (*n* = 76; 86%), and 12 (14%) reported that there was at least one conflict of interest. The content of these 12 statements is summarized in Table 3.

### Replication and evidence synthesis

Of the 188 articles involving primary or secondary data, 10 (5%, 95% CI = [3%, 8%]) claimed to include a replication study. Of the 183 articles involving primary data, one article (1%, 95% CI = [0%, 1%]) was cited by another article that claimed to be a replication. Of the 183 articles involving primary data, 21 were formally included in systematic reviews (11%, 95% CI = [8%, 16%]). An additional eight articles were cited incidentally (there was no intention to include in the review) but not formally included, and one additional article was explicitly excluded. Twelve articles were formally included in meta-analyses (7%, 95% CI = [4%, 10%]). Two additional articles were cited incidentally but not formally included, and one additional article was excluded. Meta-analyses and systematic reviews were coded as exclusive variables (such that if a systematic review included a meta-analysis, we coded it as a meta-analysis and not a systematic review); thus, the 12 meta-analyses and 21 systematic reviews sum to a total of 33 evidence-synthesis articles. Overall, the 228 English-language and accessible articles tended to be infrequently cited (*Mdn* = 3; minimum = 0; maximum = 74).

## Discussion

Our evaluation of transparency and reproducibility-related research practices in a random sample of 250 psychology articles published between 2014 and 2017 shows that, although many articles were publicly available, crucial components of research—protocols, materials, raw data, and analysis scripts—were rarely made publicly available alongside them. Preregistration remained a nascent proposition with minimal adoption. The disclosure of funding sources and conflicts of interest was modest. Replication or evidence synthesis via meta-analysis or systematic review was infrequent (although, admittedly, only a relatively short time had elapsed since the articles had been published). Although there is evidence that some individual methodological reform initiatives have been effective in specific situations (e.g., Hardwicke et al., 2018; Nuijten et al., 2017; for review, see Hardwicke, Serghiou, et al., 2020), the findings of the current study imply that their collective, broader impact on the psychology literature during the examined period was still fairly limited in scope.

For most of the articles (65%) we examined, we could access a publicly available version (open access). This is higher than recent open-access estimates obtained for biomedicine (25%; Wallach et al., 2018) and the social sciences (40%; Hardwicke, Wallach, et al., 2020), as well as a large-scale automated analysis that suggested that 45% of the scientific literature published in 2015 was publicly available (Piwowar et al., 2018). Limiting access to academic publications reduces opportunities for researchers, policymakers, practitioners, and the general public to evaluate and make use of scientific evidence. One step psychologists can take to improve the public availability of their articles is to upload them to the free preprint server PsyArXiv (https://psyarxiv.com/). Uploading a preprint does not preclude publication at most journals (Bourne et al., 2017), although specific policies regarding open access can be checked on the Sherpa/Romeo database (http://sherpa.ac.uk/romeo/index.php).

The reported availability of research materials was modest in the articles we examined (14%), which is comparable to recent estimates in the social sciences (11%; Hardwicke, Wallach, et al., 2020) and lower than in biomedicine (33%; Wallach et al., 2018). Several reportedly available sets of materials were in fact not available because of broken links, an example of the “link-rot” phenomenon that has been observed by others trying to access research resources (Evangelou et al., 2005; Rowhani-Farid & Barnett, 2018). We also did not find any study protocols (an additional document detailing the study methods); however, it is unclear to what extent this results from a difference in norms between, for example, biomedicine (in which prespecified protocols are increasingly promoted; Ioannidis, Greenland, et al., 2014) and psychology (in which there may not be an expectation to provide methodological details in a separate protocol document). We did not examine whether sufficient methodological information was provided in the Method sections of articles, as this would have required domain-specific expertise in the many topics addressed by the articles in our sample. The availability of original research materials (e.g., survey instruments, stimuli, software, videos) and protocols enables the comprehensive evaluation of research (during traditional peer review and beyond; Vazire, 2017) and high-fidelity independent replication attempts (Open Science Collaboration, 2015; Simons, 2014), both of which are important for the verification and systematic accumulation of scientific knowledge (Ioannidis, 2012). Furthermore, reusing materials and protocols reduces waste and enhances efficiency (Chalmers & Glasziou, 2009; Ioannidis, Greenland, et al., 2014). Psychologists can share their materials and protocols online in various third-party repositories that use stable permalinks, such as the Open Science Framework^2^ (OSF; see Klein et al., 2018). One observational study found that when the journal *Psychological Science* offered authors an open-materials badge there was a subsequent increase in the sharing of materials (Kidwell et al., 2016).

Data-availability statements in the articles we examined were extremely uncommon. This is consistent with accumulating evidence that suggests that the data underlying scientific claims are rarely immediately available (Alsheikh-Ali et al., 2011; Iqbal et al., 2016), although some modest improvement has been observed in recent years in biomedicine (Wallach et al., 2018). Although we did not request data from authors directly, such requests to psychology researchers typically have a modest yield (Vanpaemel et al., 2015; Wicherts et al., 2006). Most data appear to be effectively lost, including for some of the most influential studies in psychology and psychiatry (Hardwicke & Ioannidis, 2018b). Vanpaemel et al. (2015), for example, could not obtain 62% of the 394 data sets they requested from authors of papers published in four American Psychological Association journals in 2012. The sharing of raw data, which is the evidence on which scientists base their claims, enables verification through the independent assessment of analytic or computational reproducibility (Hardwicke, Bohn, et al., 2020; Hardwicke et al., 2018; LeBel et al., 2018) and analytic robustness (Steegen et al., 2016). Data sharing also enhances evidence synthesis, such as through individual participant-level meta-analysis (Tierney et al., 2015), and can facilitate discovery, such as through the merging of data sets and reanalysis with novel techniques (Voytek, 2016). Psychologists can improve data availability by uploading raw data to third-party repositories such as the OSF (Klein et al., 2018). Data sharing must be managed with caution if there are ethical concerns, but such concerns do not always preclude all forms of sharing or necessarily negate ethical motivations for sharing (Meyer, 2017). Furthermore, when data cannot be made available it is always possible to explicitly declare this in research articles and explain the rationale for not sharing (Morey et al., 2016). Journal policies that use badges to encourage data sharing (Kidwell et al., 2016) or mandate data sharing (Hardwicke et al., 2018; Nuijten et al., 2017) have been associated with marked increases in data availability in the journals that adopted them.

Of the articles we examined, only one shared an analysis script, a dearth consistent with assessments in biomedicine (Wallach et al., 2018), the social sciences (Hardwicke, Wallach, et al., 2020), and biostatistics (Rowhani-Farid & Barnett, 2018). Analysis scripts (a step-by-step description of the analysis in the form of computer code or instructions for recreating the analysis in point-and-click software) provide the most veridical documentation of how the raw data were filtered, summarized, and analyzed. Verbal descriptions of analysis procedures are often ambiguous, contain errors, or do not adequately capture sufficient detail to enable analytic reproducibility (Hardwicke, Bohn, et al., 2020; Hardwicke et al., 2018; Stodden et al., 2018). Psychologists can share their analysis scripts on a third-party repository, such as the OSF (Klein et al., 2018), and educational resources are available to help researchers improve the quality of their analysis code (Wilson et al., 2017). Sharing the computational environment in which analysis code successfully runs may also help to promote its longevity and trouble-free transfer to other researchers’ computers (Clyburne-Sherin et al., 2018).

Preregistration, which involves making a time-stamped, read-only record of a study’s rationale, hypotheses, methods, and analysis plan on an independent online repository, was rare in the articles we examined. Preregistration fulfills a number of potential functions (Nosek et al., 2019), including clarifying the distinction between exploratory and confirmatory aspects of research (Kimmelman et al., 2014; Wagenmakers et al., 2012) and enabling the detection and mitigation of questionable research practices such as selective-outcome reporting (Franco et al., 2016; John et al., 2012; Simmons et al., 2011). Preregistration is relatively new to psychology (Nosek et al., 2018, 2019), but similar concepts of registration have a longer history in the context of clinical trials in biomedicine (Dickersin & Rennie, 2012), in which they have become the expected norm (Zarin et al., 2017). However, clinical trials represent only a minority of biomedical research, and estimates suggest that preregistration is rare in biomedicine overall (Iqbal et al., 2016; Wallach et al., 2018). Preregistration is also rare in the social sciences (Hardwicke, Wallach, et al., 2020). There is no doubt that the number of preregistrations (and the related Registered Reports article format) is increasing in psychology (Hardwicke & Ioannidis, 2018a; Nosek et al., 2018); however, our findings suggest that efforts to promote preregistration may not yet have had widespread impact on routine practice. It is important to note that because there is a time lag between registration and study publication, our measures may underestimate adoption. Although norms and standards for preregistration in psychology are still evolving (Nosek et al., 2019), several dedicated registries, such as the OSF, will host preregistrations, and detailed guidance is available (Klein et al., 2018).

Our findings suggest that psychology articles were more likely to include funding statements (62%) and conflict-of-interest statements (39%) than social-science articles in general (31% and 15%, respectively; Hardwicke, Wallach, et al., 2020) but less likely than biomedical articles (69% and 65%, respectively; Wallach et al., 2018). It is possible that these disclosure statements are more common than most other practices we examined because they are often mandated by journals (Nutu et al., 2019). Disclosing funding sources and potential conflicts of interest in research articles helps readers to make informed judgments about the risk of bias (Bekelman et al., 2003; Cristea & Ioannidis, 2018). In the absence of established norms or journal mandates, authors may often assume that such statements are not relevant to them (Chivers, 2019). However, because the absence of a statement is ambiguous, researchers should ideally always include one, even if it is to explicitly declare that there were no funding sources and no potential conflicts of interest.

Of the articles we examined, 5% claimed to be a replication study—slightly higher than a previous estimate in psychology of 1% (Makel et al., 2012) and a similar estimate of 1% in the social sciences (Hardwicke, Wallach, et al., 2020) but comparable to a 5% estimate in biomedicine (Wallach et al. 2018). Only 1% of the articles we examined were cited by another article that claimed to be a replication attempt; of these articles, 11% were included in a systematic review, and 7% were included in a meta-analysis. Replication and evidence synthesis through systematic reviews and meta-analyses help to verify and build on the existing evidence base. However, it is unclear what an ideal frequency of these activities would be because they depend on many factors, such as how often studies are sufficiently similar to be amenable to synthesis methods. Although the current findings suggest that routine replication and evidence synthesis is relatively rare in psychology, many high-profile replication attempts have been conducted in recent years (Open Science Collaboration, 2015; Pashler & Wagenmakers, 2012). In addition, because the articles we examined were published relatively recently, there may be some time lag before relevant replication and evidence-synthesis studies emerge. For example, in biomedicine at least, there is a geometric growth in the number of meta-analyses, and in many fields multiple meta-analyses are often conducted once several studies appear on the same research question (Ioannidis, 2016).

The current study has several caveats and limitations. First, our findings are based on a random sample of 250 articles, and the obtained estimates may not necessarily generalize to specific contexts, such as other disciplines, subfields of psychology, or articles published in particular journals. However, this target sample size was selected to balance informativeness with tractability, and the observed estimates have reasonable precision. Second, although the focus of this study was transparency and reproducibility-related practices, this does not imply that the adoption of these practices is sufficient to promote the goals they are intended to achieve. For example, poorly documented data may not enable analytic reproducibility (Hardwicke, Bohn, et al., 2020; Hardwicke et al., 2018), and inadequately specified preregistrations may not sufficiently constrain researcher degrees of freedom (Claesen et al., 2019; Bakker et al., 2020). Third, we relied only on published information. Direct requests to authors may have yielded additional information; however, as noted earlier, such requests to research psychologists are often unsuccessful (Hardwicke & Ioannidis, 2018a; Vanpaemel et al., 2015; Wicherts et al., 2006). Fourth, a lack of transparency may have been justified in some cases if there were overriding practical, legal, or ethical concerns (Meyer, 2017). However, no constraints of this kind were declared in any of the articles we examined. Last, the study can gauge the prevalence of the assessed practices only during a particular time period. The effect of reform initiatives introduced after the examined time period, such as the founding of the Society for Improving Psychological Science (http://improvingpsych.org), will not be represented in our findings.

The current findings imply the minimal adoption of transparency and reproducibility-related practices in psychology during the examined time period. Although researchers appear to recognize the problems of low credibility and reproducibility (Baker, 2016) and endorse the values of transparency and reproducibility in principle (Anderson et al., 2010), they are often wary of change (Fuchs et al., 2012; Houtkoop et al., 2018) and routinely neglect these principles in practice (Hardwicke, Wallach, et al., 2020; Iqbal et al., 2016; Wallach et al., 2018). There is unlikely to be a single remedy to this situation. A multifaceted approach will likely be required, with iterative evaluation and careful scrutiny of reform initiatives (Hardwicke, Serghiou, et al., 2020). At the educational level, guidance and resources are available to aid researchers (Crüwell et al., 2019; Klein et al., 2018). At the institutional level, there is evidence that funder and journal policies can be effective at fomenting change (Hardwicke et al., 2018; Nuijten et al., 2017), and these initiatives should be translated and disseminated where relevant. Heterogeneous journal policies (Nutu et al., 2019) may currently be disrupting efforts to establish norms and promote better standards in routine practice. The Transparency and Openness Promotion initiative promises to encourage the adoption and standardization of journal policies related to transparency and reproducibility (Nosek et al., 2015), but it remains to be seen how effective this initiative will be in practice. Aligning academic rewards and incentives (e.g., funding awards, publication acceptance, promotion, and tenure) with better research practices may also be instrumental in encouraging wider adoption of these practices (Moher et al., 2018).

The current study is one of several to examine the prevalence of transparency and reproducibility-related research practices across scientific disciplines (Hardwicke, Wallach, et al., 2020; Iqbal et al., 2016; Wallach et al., 2018). Here, we have sketched out some of the topography of psychology’s territory. Additional studies will be required to fill in areas of the map that have yet to be explored and increase the resolution in specific areas (e.g., subfields of psychology). Future studies can also add a temporal dimension by comparing new data with the baseline established here, allowing us to explore the evolution of this landscape over time.

## Supplemental Material

sj-pdf-1-pps-10.1177_1745691620979806 – Supplemental material for Estimating the Prevalence of Transparency and Reproducibility-Related Research Practices in Psychology (2014–2017) Click here for additional data file. Supplemental material, sj-pdf-1-pps-10.1177_1745691620979806 for Estimating the Prevalence of Transparency and Reproducibility-Related Research Practices in Psychology (2014–2017) by Tom E. Hardwicke, Robert T. Thibault, Jessica E. Kosie, Joshua D. Wallach, Mallory C. Kidwell and John P. A. Ioannidis in Perspectives on Psychological Science

## Transparency

*Action Editor:* Richard Lucas

*Editor:* Laura A. King

*Author Contributions*

T. E. Hardwicke, J. D. Wallach, and J. P. A. Ioannidis designed the study; T. E. Hardwicke, J. D. Wallach, M. C. Kidwell, R. T. Thibault, and J. E. Kosie collected the data; T. E. Hardwicke analyzed the data; and T. E. Hardwicke, J. D. Wallach, R. T. Thibault, and J. P. A. Ioannidis wrote the manuscript. All of the authors approved the final manuscript for submission.

## Notes

1. Although in some domains, such as biomedicine, there is an expectation that the study objectives, design, and methods will be detailed in a prespecified document called a protocol (Ioannidis, Greenland, et al., 2014), the prevailing expectation in psychology may be that this information is fully outlined in the final research report and not necessarily prespecified in a separate document. To enable a comparison with similar studies conducted in the biomedical domain (Iqbal et al., 2016; Wallach et al., 2018), we have retained the protocol variable but acknowledge it may be less meaningful in the context of psychological research (see Discussion).

2. We mention the OSF only in the interests of brevity. However, other repositories are available that offer similar functionality for storing and sharing some research resources (e.g., Inter-University Consortium for Political and Social Research, Dataverse, Odum Institute, TalkBank, OpenNeuro, and Databrary). For a detailed discussion, see Klein et al. (2018).

## References

[bibr1-1745691620979806] Alsheikh-Ali A. A. Qureshi W. Al-Mallah M. H. Ioannidis J. P. A. (2011). Public availability of published research data in high-impact journals. PLOS ONE, 6(9), Article e24357. 10.1371/journal.pone.0024357 PMC3168487 21915316

[bibr2-1745691620979806] Anderson M. S. Ronning E. A. Devries R. Martinson B. C. (2010). Extending the Mertonian norms: Scientists’ subscription to norms of research. The Journal of Higher Education, 81(3), 366–393. 10.1353/jhe.0.0095 21132074 PMC2995462

[bibr3-1745691620979806] Baker M. (2016). 1,500 scientists lift the lid on reproducibility. Nature, 533(7604), 452–454. 10.1038/533452a 27225100

[bibr4-1745691620979806] Bakker M. Veldkamp C. L. S. van Assen M. A. L. M. Crompvoets E. A. V. Ong H. H. Nosek B. A. Soderberg C. K. Mellor D. Wicherts J. M. (2020). Ensuring the quality and specificity of preregistrations. PLOS Biology, 18(12), Article e3000937. 10.1371/journal.pbio.3000937 PMC7725296 33296358

[bibr5-1745691620979806] Bekelman J. E. Li Y. Gross C. P. (2003). Scope and impact of financial conflicts of interest in biomedical research: A systematic review. JAMA, 289(4), 454–465. 10.1001/jama.289.4.454 12533125

[bibr6-1745691620979806] Bourne P. E. Polka J. K. Vale R. D. Kiley R. (2017). Ten simple rules to consider regarding preprint submission. PLOS Computational Biology, 13(5), Article 1005473. 10.1371/journal.pcbi.1005473 PMC5417409 28472041

[bibr7-1745691620979806] Buckheit J. B. Donoho D. L. (1995). WaveLab and reproducible research. In Bickel P. Diggle P. Fienberg S. Krickeberg K. Olkin I. Wermuth N. Zeger S. Antoniadis A. Oppenheim G. (Eds.), Wavelets and statistics (Vol. 103, pp. 55–81). Springer. 10.1007/978-1-4612-2544-7_5

[bibr8-1745691620979806] Chalmers I. Glasziou P. (2009). Avoidable waste in the production and reporting of research evidence. Lancet, 374(9683), 86–89. 10.1016/s0140-6736(09)60329-9 19525005

[bibr9-1745691620979806] Chivers T. (2019). Does psychology have a conflict-of-interest problem? Nature, 571(7763), 20–23. 10.1038/d41586-019-02041-5 31267062

[bibr10-1745691620979806] Claesen A. Gomes S. Tuerlinckx F. Vanpaemel W. (2019). Preregistration: Comparing dream to reality. PsyArXiv. 10.31234/osf.io/d8wex PMC8548785 34729209

[bibr11-1745691620979806] Clyburne-Sherin A. Fei X. Green S. A. (2018). Computational reproducibility via containers in psychology. PsyArXiv. 10.31234/osf.io/mf82t

[bibr12-1745691620979806] Cristea I.-A. Ioannidis J. P. A. (2018). Improving disclosure of financial conflicts of interest for research on psychosocial interventions. JAMA Psychiatry, 75(6), 541–542. 10.1001/jamapsychiatry.2018.0382 29641818

[bibr13-1745691620979806] Crüwell S. van Doorn J. Etz A. Makel M. C. Moshontz H. Niebaum J. Orben A. Parsons S. Schulte-Mecklenbeck M. (2019). Seven easy steps to open science: An annotated reading list. Zeitschrift für Psychologie, 227(4), 237–248. 10.1027/2151-2604/a000387

[bibr14-1745691620979806] David P. A. (2008). The historical origins of ‘Open Science’: An essay on patronage, reputation and common agency contracting in the scientific revolution. Capitalism and Society, 3(2). 10.2202/1932-0213.1040

[bibr15-1745691620979806] Dickersin K. Rennie D. (2012). The evolution of trial registries and their use to assess the clinical trial enterprise. JAMA, 307(17), 1861–1864. 10.1001/jama.2012.4230 22550202

[bibr16-1745691620979806] Evangelou E. Trikalinos T. A. Ioannidis J. P. A. (2005). Unavailability of online supplementary scientific information from articles published in major journals. FASEB Journal, 19(14), 1943–1944. 10.1096/fj.05-4784lsf 16319137

[bibr17-1745691620979806] Franco A. Malhotra N. Simonovits G. (2016). Underreporting in psychology experiments. Social Psychological and Personality Science, 7(1), 8–12. 10.1177/1948550615598377

[bibr18-1745691620979806] Fuchs H. M. Jenny M. Fiedler S. (2012). Psychologists are open to change, yet wary of rules. Perspectives on Psychological Science, 7(6), 639–642. 10.1177/1745691612459521 26168123

[bibr19-1745691620979806] Hardwicke T. E. Bohn M. MacDonald K. E. Hembacher E. Nuijten M. B. Peloquin B. deMayo B. E. Long B. Yoon E. J. Frank M. C. (2020). Analytic reproducibility in articles receiving open data badges at the journal *Psychological Science*: An observational study. Royal Society Open Science, 8, Article 201494. 10.1098/rsos.201494 PMC7890505 33614084

[bibr20-1745691620979806] Hardwicke T. E. Ioannidis J. P. A. (2018a). Mapping the universe of registered reports. Nature Human Behaviour, 2(11), 793–796. 10.1038/s41562-018-0444-y 31558810

[bibr21-1745691620979806] Hardwicke T. E. Ioannidis J. P. A. (2018b). Populating the Data Ark: An attempt to retrieve, preserve, and liberate data from the most highly-cited psychology and psychiatry articles. PLOS ONE, 13(8), Article e0201856. 10.1371/journal.pone.0201856 PMC6072126 30071110

[bibr22-1745691620979806] Hardwicke T. E. Mathur M. B. MacDonald K. Nilsonne G. Banks G. C. Kidwell M. C. Mohr A. H. Clayton E. Yoon E. J. Tessler M. H. Lenne R. L. Altman S. Long B. Frank M. C. (2018). Data availability, reusability, and analytic reproducibility: Evaluating the impact of a mandatory open data policy at the journal *Cognition*. Royal Society Open Science, 5(8), Article 180448. 10.1098/rsos.180448 PMC6124055 30225032

[bibr23-1745691620979806] Hardwicke T. E. Serghiou S. Janiaud P. Danchev V. Crüwell S. Goodman S. Ioannidis J. P. A. (2020). Calibrating the scientific ecosystem through meta-research. Annual Review of Statistics and Its Application, 7, 11–37. 10.1146/annurev-statistics-031219-041104

[bibr24-1745691620979806] Hardwicke T. E. Wallach J. D. Kidwell M. Bendixen T. Crüwell S. Ioannidis J. P. A. (2020). An empirical assessment of transparency and reproducibility-related research practices in the social sciences (2014–2017). Royal Society Open Science, 7(2), Article 190806. 10.1098/rsos.190806 PMC7062098 32257301

[bibr25-1745691620979806] Houtkoop B. L. Chambers C. Macleod M. Bishop D. V. M. Nichols T. E. Wagenmakers E.-J. (2018). Data sharing in psychology: A survey on barriers and preconditions. Advances in Methods and Practices in Psychological Science, 1(1), 70–85. 10.1177/2515245917751886

[bibr26-1745691620979806] Ioannidis J. P. A. (2005). Why most published research findings are false. PLOS Medicine, 2(8), Article e124. 10.1371/journal.pmed.0020124 PMC1182327 16060722

[bibr27-1745691620979806] Ioannidis J. P. A. (2012). Why science is not necessarily self-correcting. Perspectives on Psychological Science, 7(6), 645–654. 10.1177/1745691612464056 26168125

[bibr28-1745691620979806] Ioannidis J. P. A. (2016). The mass production of redundant, misleading, and conflicted systematic reviews and meta-analyses: Mass production of systematic reviews and meta-analyses. The Milbank Quarterly, 94(3), 485–514. 10.1111/1468-0009.12210 27620683 PMC5020151

[bibr29-1745691620979806] Ioannidis J. P. A. Greenland S. Hlatky M. A. Khoury M. J. Macleod M. R. Moher D. Schulz K. F. Tibshirani R. (2014). Increasing value and reducing waste in research design, conduct, and analysis. Lancet, 383(9912), 166–175. 10.1016/s0140-6736(13)62227-8 24411645 PMC4697939

[bibr30-1745691620979806] Ioannidis J. P. A. Munafò M. R. Fusar-Poli P. Nosek B. A. David S. P. (2014). Publication and other reporting biases in cognitive sciences: Detection, prevalence, and prevention. Trends in Cognitive Sciences, 18(5), 235–241. 10.1016/j.tics.2014.02.010 24656991 PMC4078993

[bibr31-1745691620979806] Iqbal S. A. Wallach J. D. Khoury M. J. Schully S. D. Ioannidis J. P. A. (2016). Reproducible research practices and transparency across the biomedical literature. PLOS Biology, 14(1), Article e1002333. 10.1371/journal.pbio.1002333 PMC4699702 26726926

[bibr32-1745691620979806] John L. K. Loewenstein G. Prelec D. (2012). Measuring the prevalence of questionable research practices with incentives for truth telling. Psychological Science, 23(5), 524–532. 10.1177/0956797611430953 22508865

[bibr33-1745691620979806] Kidwell M. C. Lazarevic L. B. Baranski E. Hardwicke T. E. Piechowski S. Falkenberg L.-S. Kennett C. Slowik A. Sonnleitner C. Hess-Holden C. Errington T. M. Fiedler S. Nosek B. A. (2016). Badges to acknowledge open practices: A simple, low-cost, effective method for increasing transparency. PLOS Biology, 14(5), Article e1002456. 10.1371/journal.pbio.1002456 PMC4865119 27171007

[bibr34-1745691620979806] Kimmelman J. Mogil J. S. Dirnagl U. (2014). Distinguishing between exploratory and confirmatory preclinical research will improve translation. PLOS Biology, 12(5), Article e1001863. 10.1371/journal.pbio.1001863 PMC4028181 24844265

[bibr35-1745691620979806] Klein O. Hardwicke T. E. Aust F. Breuer J. Danielsson H. Mohr A. H. Ijzerman H. Nilsonne G. Vanpaemel W. Frank M. C. (2018). A practical guide for transparency in psychological science. Collabra: Psychology, 4(1), Article 20. 10.1525/collabra.158

[bibr36-1745691620979806] LeBel E. P. McCarthy R. J. Earp B. D. Elson M. Vanpaemel W. (2018). A unified framework to quantify the credibility of scientific findings. Advances in Methods and Practices in Psychological Science, 1(3), 389–402. 10.1177/2515245918787489

[bibr37-1745691620979806] Makel M. C. Plucker J. A. Hegarty B. (2012). Replications in psychology research. Perspectives on Psychological Science, 7(6), 537–542. 10.1177/1745691612460688 26168110

[bibr38-1745691620979806] Meyer M. N. (2017). Practical tips for ethical data sharing. Advances in Methods and Practices in Psychological Science, 1(1), 131–144. 10.1177/2515245917747656

[bibr39-1745691620979806] Miguel E. Camerer C. Casey K. Cohen J. Esterling K. M. Gerber A. Glennerster R. Green D. P. Humphreys M. Imbens G. Laitin D. Madon T. Nelson L. Nosek B. A. Petersen M. Sedlmayr R. Simmons J. P. Simonsohn U. der Laan M. V. (2014). Promoting transparency in social science research. Science, 343(6166), 30–31. 10.1126/science.1245317 24385620 PMC4103621

[bibr40-1745691620979806] Moher D. Naudet F. Cristea I. A. Miedema F. Ioannidis J. P. A. Goodman S. N. (2018). Assessing scientists for hiring, promotion, and tenure. PLOS Biology, 16, Article e2004089. 10.1371/journal.pbio.2004089 PMC5892914 29596415

[bibr41-1745691620979806] Morey R. D. Chambers C. D. Etchells P. J. Harris C. R. Hoekstra R. Lakens D. Lewandowsky S. Morey C. C. Newman D. P. Schönbrodt F. D. Vanpaemel W. Wagenmakers E.-J. Zwaan R. A. (2016). The Peer Reviewers’ Openness Initiative: Incentivizing open research practices through peer review. Royal Society Open Science, 3(1), Article 150547. 10.1098/rsos.150547 PMC4736937 26909182

[bibr42-1745691620979806] Munafò M. R. Nosek B. A. Bishop D. V. M. Button K. S. Chambers C. D. du Sert N. P. Simonsohn U. Wagenmakers E.-J. Ware J. J. Ioannidis J. P. A. (2017). A manifesto for reproducible science. Nature Human Behaviour, 1(1), 1–9. 10.1038/s41562-016-0021 PMC7610724 33954258

[bibr43-1745691620979806] Naudet F. Sakarovitch C. Janiaud P. Cristea I. Fanelli D. Moher D. Ioannidis J. P. A. (2018). Data sharing and reanalysis of randomized controlled trials in leading biomedical journals with a full data sharing policy: Survey of studies published in *The BMJ* and *PLOS Medicine*. BMJ, 360, Article k400. 10.1136/bmj.k400 PMC5809812 29440066

[bibr44-1745691620979806] Nelson L. D. Simmons J. P. Simonsohn U. (2018). Psychology’s renaissance. Annual Review of Psychology, 69(1), 511–534. 10.1146/annurev-psych-122216-011836 29068778

[bibr45-1745691620979806] Nosek B. A. Alter G. Banks G. C. Borsboom D. Bowman S. D. Breckler S. J. Buck S. Chambers C. D. Chin G. Christensen G. Contestabile M. Dafoe A. Eich E. Freese J. Glennerster R. Goroff D. Green D. P. Hesse B. Humphreys M. . . . Yarkoni T. (2015). Promoting an open research culture. Science, 348(6242), 1422–1425. 10.1126/science.aab2374 26113702 PMC4550299

[bibr46-1745691620979806] Nosek B. A. Beck E. D. Campbell L. Flake J. K. Hardwicke T. E. Mellor D. T. van ’t Veer A. E. Vazire S. (2019). Preregistration is hard, and worthwhile. Trends in Cognitive Sciences, 23(10), P815–P818. 10.1016/j.tics.2019.07.009 31421987

[bibr47-1745691620979806] Nosek B. A. Ebersole C. R. DeHaven A. C. Mellor D. T. (2018). The preregistration revolution. Proceedings of the National Academy of Sciences, USA, 115(11), 2600–2606. 10.1073/pnas.1708274114 PMC5856500 29531091

[bibr48-1745691620979806] Nuijten M. B. Borghuis J. Veldkamp C. L. S. Dominguez-Alvarez L. van Assen M. A. L. M. Wicherts J. M. (2017). Journal data sharing policies and statistical reporting inconsistencies in psychology. Collabra: Psychology, 3(1), Article 31. 10.1525/collabra.102

[bibr49-1745691620979806] Nutu D. Gentili C. Naudet F. Cristea I. A. (2019). Open science practices in clinical psychology journals: An audit study. Journal of Abnormal Psychology, 128(6), 510–516. 10.1037/abn0000414 31368730

[bibr50-1745691620979806] Open Science Collaboration. (2015). Estimating the reproducibility of psychological science. Science, 349(6251), Article aac4716. 10.1126/science.aac4716 26315443

[bibr51-1745691620979806] Pashler H. Wagenmakers E.-J. (2012). Editors’ introduction to the special section on replicability in psychological science. Perspectives on Psychological Science, 7(6), 528–530. 10.1177/1745691612465253 26168108

[bibr52-1745691620979806] Piwowar H. Priem J. Lariviere V. Alperin J. P. Matthias L. Norlander B. Farley A. West J. Haustein S. (2018). The state of OA: A large-scale analysis of the prevalence and impact of Open Access articles. PeerJ, 6, Article e4375. 10.7717/peerj.4375 PMC5815332 29456894

[bibr53-1745691620979806] Rowhani-Farid A. Barnett A. G. (2016). Has open data arrived at the *British Medical Journal (BMJ)*? An observational study. BMJ Open, 6(10), Article e011784. 10.1136/bmjopen-2016-011784 PMC5073489 27737882

[bibr54-1745691620979806] Rowhani-Farid A. Barnett A. G. (2018). Badges for sharing data and code at Biostatistics: An observational study. F1000Research, 7, Article 90. 10.12688/f1000research.13477.2 PMC5843843 29862016

[bibr55-1745691620979806] Simmons J. P. Nelson L. D. Simonsohn U. (2011). False-positive psychology: Undisclosed flexibility in data collection and analysis allows presenting anything as significant. Psychological Science, 22(11), 1359–1366. 10.1177/0956797611417632 22006061

[bibr56-1745691620979806] Simons D. J. (2014). The value of direct replication. Perspectives on Psychological Science, 9(1), 76–80. 10.1177/1745691613514755 26173243

[bibr57-1745691620979806] Sison C. P. Glaz J. (1995). Simultaneous confidence intervals and sample size determination for multinomial proportions. Journal of the American Statistical Association, 90(429), 366–369. 10.1080/01621459.1995.10476521

[bibr58-1745691620979806] Steegen S. Tuerlinckx F. Gelman A. Vanpaemel W. (2016). Increasing transparency through a multiverse analysis. Perspectives on Psychological Science, 11(5), 702–712. 10.1177/1745691616658637 27694465

[bibr59-1745691620979806] Stodden V. Seiler J. Ma Z. (2018). An empirical analysis of journal policy effectiveness for computational reproducibility. Proceedings of the National Academy of Sciences, USA, 115(11), Article 201708290. 10.1073/pnas.1708290115 PMC5856507 29531050

[bibr60-1745691620979806] Szucs D. Ioannidis J. P. A. (2017). Empirical assessment of published effect sizes and power in the recent cognitive neuroscience and psychology literature. PLOS Biology, 15(3), Article e2000797. 10.1371/journal.pbio.2000797 PMC5333800 28253258

[bibr61-1745691620979806] Tierney J. F. Vale C. Riley R. Smith C. T. Stewart L. Clarke M. Rovers M. (2015). Individual Participant Data (IPD) meta-analyses of randomised controlled trials: Guidance on their use. PLOS Medicine, 12(7), Article e1001855. 10.1371/journal.pmed.1001855 PMC4510878 26196287

[bibr62-1745691620979806] Vanpaemel W. Vermorgen M. Deriemaecker L. Storms G. (2015). Are we wasting a good crisis? The availability of psychological research data after the storm. Collabra: Psychology, 1(1), Article 3. 10.1525/collabra.13

[bibr63-1745691620979806] Vazire S. (2017). Quality uncertainty erodes trust in science. Collabra: Psychology, 3(1), Article 1. 10.1525/collabra.74

[bibr64-1745691620979806] Vazire S. (2018). Implications of the credibility revolution for productivity, creativity, and progress. Perspectives on Psychological Science, 13(4), 411–417. 10.1177/1745691617751884 29961410

[bibr65-1745691620979806] Voytek B. (2016). The virtuous cycle of a data ecosystem. PLOS Computational Biology, 12(8), Article e1005037. 10.1371/journal.pcbi.1005037 PMC4974004 27490108

[bibr66-1745691620979806] Wagenmakers E.-J. Wetzels R. Borsboom D. van der Maas H. L. J. Kievit R. A. (2012). An agenda for purely confirmatory research. Perspectives on Psychological Science, 7(6), 632–638. 10.1177/1745691612463078 26168122

[bibr67-1745691620979806] Wallach J. D. Boyack K. W. Ioannidis J. P. A. (2018). Reproducible research practices, transparency, and open access data in the biomedical literature, 2015-2017. PLOS Biology, 16(11), Article e2006930. 10.1371/journal.pbio.2006930 PMC6245499 30457984

[bibr68-1745691620979806] Wallach J. D. Gonsalves G. S. Ross J. S. (2018). Research, regulatory, and clinical decision-making: The importance of scientific integrity. Journal of Clinical Epidemiology, 93, 88–93. 10.1016/j.jclinepi.2017.08.021 29042327

[bibr69-1745691620979806] Wicherts J. M. Borsboom D. Kats J. Molenaar D. (2006). The poor availability of psychological research data for reanalysis. American Psychologist, 61(7), 726–728. 10.1037/0003-066x.61.7.726 17032082

[bibr70-1745691620979806] Wilson G. Bryan J. Cranston K. Kitzes J. Nederbragt L. Teal T. K. (2017). Good enough practices in scientific computing. PLOS Computational Biology, 13(6), Article e1005510. 10.1371/journal.pcbi.1005510 PMC5480810 28640806

[bibr71-1745691620979806] Zarin D. A. Tse T. Williams R. J. Rajakannan T. (2017). Update on trial registration 11 years after the ICMJE policy was established. The New England Journal of Medicine, 4, 383–391. 10.1056/nejmsr1601330 PMC5813248 28121511

