# Supplementary material for: Estimating the Prevalence of Transparency and Reproducibility-Related Research Practices in Psychology (2014–2017)
Source: Perspect Psychol Sci. 2021 Mar 8;17(1):239–51. doi: 10.1177/1745691620979806 (PMC8785283; doi:10.1177/1745691620979806)
Supplement: sj-pdf-1-pps-10.1177_1745691620979806 – Supplemental material for Estimating the Prevalence of Transparency and Reproducibility-Related Research Practices in Psychology (2014–2017) [file sj-pdf-1-pps-10.1177_1745691620979806.pdf]

SUPPLEMENTAL MATERIAL

Section A. Pre-registered protocol updates

During the course of the study, we made several minor adjustments to the pre-registered protocol. These are listed below.

(1) For the questions "How does the statement indicate the materials are available?" and "How does the statement indicate the data are available?" we added an explicit response option: "Appendix within the present article".
(2) For the questions "Does the article state whether or not the materials are available?" and "Does the article state whether or not the data are available?" we added an explicit response option "Source of data/materials provided but no explicit availability statement". However, because this was introduced late in the data collection process it was not applied consistently and we have elected to re-code this response option as "No - there is no data availability statement".
(3) For the questions "What type of study is being reported?" we changed the response option 'survey' to 'survey/interview' and renamed 'field study' to 'observational study'. We did this to better match our coding decisions.
(4) For the citation evaluation, we added two questions: "How many times was the article cited *incidentally* in a meta-analysis (i.e., no intention to include in data synthesis)?" and "How many times was the article cited *incidentally* in a systematic review (i.e., no intention to include in formal review)?"
(5) For the questions "Does the article include a statement indicating whether there were funding sources?" we added the response option "Yes - the statement identifies funding sources but the private/public status is unclear"
(6) To concisely present the content of conflict of interest statements we categorized them into the types shown in Table 3. This analysis was not pre-registered.
(7) To align the extraction form with previous studies (e.g., Wallach et al., 2019) we included an additional response option ("Empirical data - cost effectiveness and/or decision analysis") for the question "What type of study is being reported?"

Section B. Inter-rater reliability

We computed inter-rater reliability using Fleiss' Kappa (see Supplementary Tables 1, 2, and 3). All coding differences were resolved through discussion.

For all variables there was substantial agreement, however, for the four variables with the lowest Fleiss' kappa (<.70), we conducted an additional inspection of the differences to explore if there were any issues of concern. In summary, few differences arose from substantive disagreement between coders and were more often due to earlier differences coding study type (which could render later variables irrelevant) or differences in how non-standard ‘other’ responses were recorded in the extraction form. Details about the differences are provided below.

Coding differences for data availability statements

For the variable “Does the article state whether or not data are available?”, there were 13 cases of coder disagreement. In two cases (wfFwp, XIWav), one coder appeared to have missed a relevant data availability statement. In two cases (bppGq, VFLFB) one coder did not search for a data availability statement because there was an earlier disagreement about how to code the study type. In 5 cases (hkNiS, KuEds, ldnNm, posTo, snvlH), one coder had used an explicit response option – “Source of data provided but no explicit availability statement” - that was introduced late in the data collection process but later re-coded to "No - there is no data availability statement" to ensure consistency (see Supplementary Information A, point 2). In one case (LaZfV) the first coder identified availability of a resource that was later determined to be materials rather than data. In one case (tIUAU), the first coder identified a statement regarding the availability of preliminary data but it was later determined that this did not pertain to the main data underlying the reported study so would be more accurately classified as "No - there is no data availability statement". In one case (VEDlR), the first coder identified a statement which said 'supplementary data' were available, but further inspection suggested that these were not data so this was not considered a data availability statement. In one case (zFYIM), both coders used the 'other' response to record that data access required a fee, but as this was a non-standardized response option, the text differed.

Coding differences for pre-registration availability statements

For the variable "Does the article state whether or not the study (or some aspect of the study) was pre-registered?", there were 5 cases of coder disagreement. In two cases (HdNmC, DfkgD), one coder appeared to have missed a relevant pre-registration availability statement. In one case (BNDMz), one coder identified a clinical trial registration number, but after discussion the article was determined to be a clinical trial protocol, rather than a completed study, and the study type was thus re-classified as "No empirical data", rendering the pre-registration variable irrelevant. In two cases (bppGq, VFLFB) one coder did not search for a pre-registration availability statement because there was an earlier disagreement about how to code the study type.

Coding differences for meta-analysis exclusions

For the variable "How many times has the article been explicitly excluded from a meta-analysis?", there were 5 cases of coder disagreement. In two cases (pxYoP, QdhZZ), one coder missed relevant information. In one case (CywSU) a coder incorrectly recorded that there was an exclusion; it was later agreed that there were none. In two cases (lvyGE, snvlH), one coder did not search for meta-analysis exclusions because there was an earlier disagreement about how to code the study type.

Coding differences for cited by a replication

For the variable "How many times has the article been cited in a replication study?", there were 6 cases of coder disagreement. In two cases (pxYoP, QdhZZ), one coder missed relevant information. In two cases (gyYOp, xOYcP), the status of a citing article as a replication was ambiguous, but following discussion the coders agreed that because the authors of those articles had not explicitly described their studies as replications, we would not classify them as such. In two cases (lvyGE, snvlH), one coder did not search for meta-analysis exclusions because there was an earlier disagreement about how to code the study type.

Table S1. Inter-rater reliability (Fleiss' Kappa) for article access and availability statements for key research resources.

Article	Materials	Protocol	Data	Analysis scripts	Pre-registration	Conflicts of interest	Funding
0.86	0.78	0.71	0.61	0.75	0.66	0.96	0.92

Table S2. Inter-rater reliability (Fleiss' Kappa) for article characteristics that were manually extracted/coded by two team members.

Country of origin	Subject type (human/animal)	Study design
1.00	0.93	0.82

Table S3. Inter-rater reliability (Fleiss' Kappa) for evidence synthesis and replication variables. MA = Meta-analysis. SR = Systematic review.

MA citations	MA exclusions	MA incidental citations	SR citations	SR exclusions	SR incidental citations	Identifies as a replication	Cited by a replication
0.92	0.66	0.75	0.84	1.00	0.82	0.71	0.49
